# Patterns and Factors Influencing Parrot (Order: Psittaciformes) Success in Establishing Thriving Naturalized Populations within the Contiguous United States

**DOI:** 10.3390/ani13132101

**Published:** 2023-06-24

**Authors:** Edwin Dickinson, Melody W. Young, Daniel Tanis, Michael C. Granatosky

**Affiliations:** 1 Department of Anatomy, College of Osteopathic Medicine, New York Institute of Technology, Old Westbury, NY 11568, USA; edicki01@nyit.edu (E.D.); myoung08@nyit.edu (M.W.Y.); dtanis@nyit.edu (D.T.); 2 Center for Biomedical Innovation, College of Osteopathic Medicine, New York Institute of Technology, Old Westbury, NY 11568, USA

**Keywords:** introduced species, brain size, monk parakeet, behavioral flexibility, urban ecology

## Abstract

**Simple Summary:**

Parrots (Order: Psittaciformes) are an ancient arboreal lineage with a long history as companion animals for humans. Since at least the 1960s, released parrots have become established in non-native ranges globally. The method of their introduction is almost certainly a direct result of the pet trade through either accidental or intentional releases. Within the continental United States there are currently seventy-three naturalized parrot species that have been observed as of May 2023. Of these, close to half have established breeding populations. The propensity for a parrot species to become established appears to be phylogenetically driven. Notably, parrots in the family Cacatuidae and Neotropical *Pyrrhua* are less successful at establishing themselves in the United States once released. We posit that what makes parrots such successful naturalized species are their charismatic nature paired with considerable intelligence and behavioral flexibility.

**Abstract:**

Parrots (Order: Psittaciformes) represent one of the most striking and ecomorphologically diverse avian clades, spanning more than two orders of magnitude in body size with populations occupying six continents. The worldwide diaspora of parrots is largely due to the pet trade, driven by human desire for bright, colorful, and intelligent animals as companions. Some introduced species have aptly inserted themselves into the local ecosystem and established successful breeding colonies all around the globe. Notably, the United States is home to several thriving populations of introduced species including red-masked parakeets (*Psittacara erythrogenys*), monk parakeets (*Myiopsitta monachus*), nanday conures (*Aratinga nenday*), and red-crowned amazons (*Amazona viridigenalis*). Their incredible success globally begs the question as to how these birds adapt so readily to novel environments. In this commentary, we trace parrots through evolutionary history, contextualize existent naturalized parrot populations within the contiguous United States, and provide a phylogenetic regression analysis of body mass and brain size based on success in establishing breeding populations. The propensity for a parrot species to become established appears to be phylogenetically driven. Notably, parrots in the family Cacatuidae and Neotropical *Pyrrhua* appear to be poor at establishing themselves in the United States once released. Although brain size among Psittaciformes did not show a significant impact on successful breeding in the continental United States, we propose that the success of parrots can be attributed to their charismatic nature, significant intelligence relative to other avian lineages, and behavioral flexibility.

## 1. Introduction: The Evolutionary History of Parrots

Parrots (Order: Psittaciformes) represent one of the most striking and ecomorphologically diverse avian clades: spanning more than two orders of magnitude in body size (~10 g in *Micropsitta* to >3.6 kg in *Strigops*) and occupying a broad array of ecological locations—from open desert to the tropical rainforests [1,2]. Along their way, parrots have conquered every continent barring Antarctica, including some of the earth’s most remote islands [1]. Accordingly, parrots encompass enormous diversity in coloration [3], call repertoires [4], and diet [5] (e.g., specialized frugivores, granivores, insectivores, nectarivores, and even omnivorous taxa with a predilection for carrion [1,5,6,7]).

The evolutionary history of parrots, and their position among avians, was of great mystery to early taxonomists [8,9,10,11]. Numerous potential sister groups were theorized, including owls (on the basis of their curved beaks), pigeons (owing to their similar humeral morphologies), and woodpeckers or cuckoos (citing their zygodactylous feet) [12]. More recently molecular phylogenies have resolved this debate, placing parrots within the Eufalconimorphae clade [10,12] and, more specifically, as the sister Order of the Passeriformes [8,9,10,11].

Birds morphologically recognizable as modern parrots are present in the fossil record by the Oligocene/Miocene boundary (~25 MYA), and by the early Miocene boundary (~20 MYA) parrots are visible within the fossil record on either side of the Pacific Ocean [13]. However, the fossil history of parrots extends far deeper than this, albeit in a shroud of hitherto unresolved controversy. Indeed, molecular studies date the divergence of parrots to ~59 MYA (51–66MYA; [14]), with the three major Neotropical parrot clades (Strigopoidea, Psittacoidea, and Cacatuoidea) emerging ~50 MYA (41–57 MYA; [14]). The earliest compelling fossil from this age is assigned to the genus *Mopsitta* by Waterhouse et al. [15] and is represented by a single, relatively large right humerus (FU 100/139) dating back to ~54 MYA recovered from the Fur Formation of Northern Denmark. However, other contemporaneous osteological remains [16] from this formation are assigned to *Rhynchaeites* (i.e., an ancestor of the modern ibis), and thus the taxonomic assignation of the FU 100/139 humerus to Psittaciformes remains ambiguous at best. Additionally, an articulated pelvis, pygostyle, and left hindlimb (UWGM 39876a) dating to 51.66 ± 0.09 MYA are known from the Green River Formation, USA [17]. This specimen, tentatively assigned to the genus *Avolatavis*, shares many features with extant parrots to the exclusion of halcyornithids and messelasturids, including a broader pelvis, wider pygostyle, and deeper trochlea cartilaginis tibialis [17]. The specimen further exhibits a robust and shortened tarsometatarsus, an inferred synapomorphy of crown parrots and their descendants. However, it remains unclear exactly where this specimen may sit within the context of early Psittaciformes and Passeriformes.

Among extant parrots, fortunately, our understanding of taxonomy is much clearer. Molecular phylogenies universally highlight the clade Strigopoidea (comprising the kea, kākā, and kākāpō) as the most basal of living Psittaciformes [11,12,18]. While these species exhibit many morphological oddities, including the slender and elongated beaks of the kea and kākā, it is important to note that these traits themselves are not considered primitive [6,7,19]. Instead, this morphology is thought to represent a specialization for sap extraction that evolved during their evolutionary isolation on the islands of New Zealand [7,19]. Subsequently, Cacatuoidea are thought to have diverged from true parrots (Psittacoidea) at ~40 MYA [20]. Numerous radiations and divergences are known within Psittacoidea, most notably the split that divides psittacids (Neotropical and Afrotropical parrots) from psittaculids (e.g., lovebirds, lories and lorikeets, tiger parrots, pygmy parrots, etc.) and the enigmatic psittrichasiids (the Pesquet’s parrot, lesser and greater vasa parrots, and the black parrots of the Seychelles and Comoros) [8]. As such, it can be inferred that all living parrots within the New World (comprising >150 neotropical species) evolved from a single common ancestor [14].

### 1.1. History of Endemic Parrots in the United States

Though no endemic parrot species currently occupy the United States, two historical populations are known to have been previously present [1]. The thick-billed parrot (*Rhyncopsitta pachyrhynca*) boasted a historical range across the American Southwest, stretching as far west as Texas and as far north as Utah [21]. However, heavy hunting pressures throughout the nineteenth century drove a sharp decline in numbers, and by the 1930s pockets of parrots existed only in Arizona and New Mexico [21,22]. The last credible sightings in each state came in 1938 (Chiricahua National Monument, AZ) and 1964 (Animas Mountains, NM), respectively [22], and though a reintroduction program was established in 1986, yielding two Arizona-born offspring in 1988, this program was written off as unsuccessful in 1993 [22]. Populations of thick-billed parrots do however persist in Western Mexico [23].

Similarly, the Carolina Parakeet (*Conuropsis carolinensis*) was once widespread across the Eastern and Central United States: with a range as far north as New York, as far south as Florida, and as far west as Colorado [24]. A recent attempt to reconstruct the extinction of this species in the wild estimated the last wild individual to have been observed around 1915 [25], with the final known captive specimen dying three years later at Cincinnati Zoo [24]. While few reliable first-hand reports of its behavior remain, attempts have been made to reconstruct the ecology of this species using genomic and geographic data [26,27,28]. For example, species distribution models from Burgio et al. [27] indicate that the two subspecies of Carolina parakeet (*C. c. carolinensis* and *C. c. ludovicianus*) occupied distinct climatic niches, with the former restricted to warmer territories of the Southeast (centered around Florida and Southern Georgia) and the coastal Carolinas. Additionally, *C. c. ludovicianus* is thought to have migrated between winter and breeding seasons, while *C. c. carolinensis* remained sedentary throughout the year. This behavior may explain how this subspecies was able to occupy such large territories across the Northern United States, where winter temperatures consistently fall well below freezing. Indeed, similar migratory behaviors are found in red-headed woodpeckers, who occupy similar ranges but migrate southeasterly away from the American Midwest during winter months [29].

### 1.2. Parrots as Naturalized Species in the United States

While the continental United States may lack endemic parrots [1], in recent decades it has become home to booming populations of non-native parrot taxa [30,31,32,33,34]. When discussing non-native species, it is important to have the correct nomenclature. Exotic species are introduced to new environments beyond their natural range. When they establish self-sustaining populations, they become naturalized. Some naturalized species become invasive, spreading rapidly and causing harm to ecosystems. Feral species are domesticated organisms that have escaped or been released into the wild and now live independently [35]. Moreover, such terms are not mutually exclusive, as a feral species may become invasive if it is able to establish and spread rapidly in a new environment [36,37].

Historically, birds have proven themselves adept at establishing themselves when introduced to new regions [34,38]. The European starling was introduced to North America in the late 19th century and has since become one of the continent’s most widespread invasive taxa [39] causing significant damage to agricultural crops [40]. Similarly, the common myna, a bird native to Asia, has become a major pest in parts of Australia, where it has caused damage to crops and infrastructure [41,42].

Parrots serve a difficult case in terms of classification as an exotic species [30,33]. While the presence of parrots in the United States is most definitely the result of the pet trade, there is no reason to suggest that parrots have been domesticated (in the strict sense of the word), thus the term feral is not appropriate [43]. As such, we will follow Uehling and colleagues [30] and use the term naturalized throughout the remainder of the manuscript. Naturalized parrots have established themselves in numerous urban and suburban environments across the globe, from North and South America to Europe and Asia [31,33,44]. In Australia, there are no non-native naturalized parrots; although there are numerous records of escapees, it remains uncertain if any individuals have successfully established themselves in the wild [45]. The presence of naturalized parrots in urban environments can have both positive and negative impacts. These birds can provide a source of beauty and enjoyment for local residents [43]. Many people enjoy watching and listening to the parrots, and some cities have even embraced them as a part of their local culture: indeed, the San Francisco Chronicle has recently decreed the red-masked parakeet (*Psittacara erythrogenys*) as the official animal of San Francisco [46,47]. On the other hand, naturalized parrots can cause problems for both human residents and local ecosystems [48,49]. These birds can be loud and disruptive, with their calls often echoing through urban areas; in addition, their large communal nests can cause damage to buildings and power lines [50]. Furthermore, naturalized parrots may compete with native bird species for resources, leading to declines in local bird populations—though these ecological impacts are currently considered minor [51].

**Figure 1:**
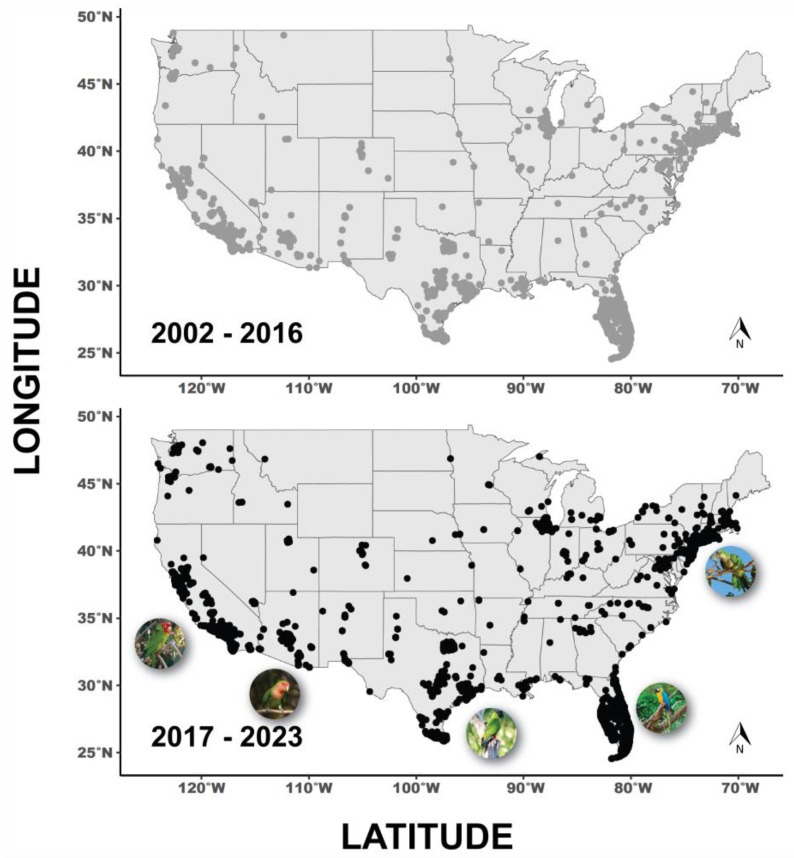
Change in distribution of unique parrot sightings between 2002–2016 (top, points in grey) recreated from Uehling and colleagues [30] (total number of observations: 141,761) and 2017–2023 (bottom, points in black; total number of observations: 332,821) in the contiguous United States. Pictured naturalized parrot species include monk parakeets (*Myiopsitta monachus*) in New York, blue-and-yellow macaws (*Ara ararauna*) in Florida, nanday conure (*Aratinga nenday*) in Texas, rosy-faced lovebirds (*Agapornis roseicollis*) in Arizona, and red-masked parakeets (*Psittacara erythrogenys*) in California. All data are from eBird Database [52].

Following the protocols of Uehling and colleagues [30], we summarized the current status (2017–2023) of naturalized parrot populations in the United States. Briefly, we downloaded records of parrot observations in the contiguous United States from eBird [52], a database run by the Cornell Lab of Ornithology (www.ebird.org; accessed on 1 May 2023). We downloaded a list of all parrot genera from TimeTree 5 [53] and then searched eBird [52] for records of any of these genera in the contiguous USA. We created two data frames: (1) between 2002–2016 to recreate the findings of Uehling and colleagues [30]; and (2) between January 2017–May 2023 to provide an updated range map of parrot species in the United States (Figure 1). Following Uehling and colleagues [30], we used parrot observations that were both “approved” and “not approved” by the eBird [52] review process. In such “not approved” instances, the precise determination of the species is typically not in doubt; instead, the entry is classified as “not approved” due to uncertainty regarding whether the species occurs naturally in the wild [52]. Thus, if there is ambiguity regarding whether a bird is wild or an escaped pet, the corresponding eBird record may be labeled as “not approved.” The criteria for approval/not approval of species exhibit some level of variation across different counties [30]. Therefore, we opted to include all verified data in our study, irrespective of whether the observations were designated as “approved” by regional reviewers. Latitude and longitude data were collected from these observations to generate a range map in R [54] using packages “geom_sf” and “ggplot2” to reflect new observations of naturalized parrot species between 2017–2023 (Table 1 and Figure 1).

**Figure 2:**
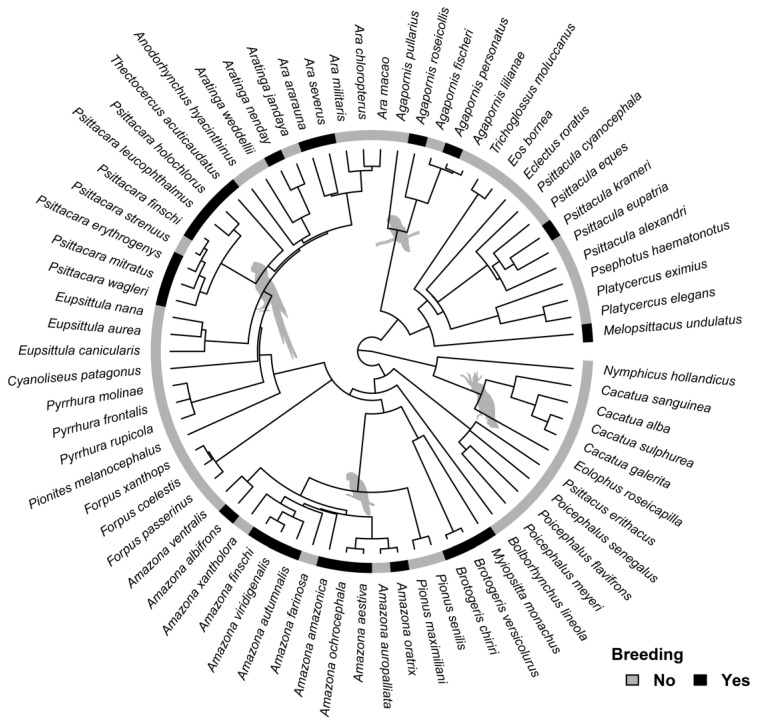
Branching phylogenetic tree showing taxonomic distribution of parrot taxa sightings from eBird database [52] between 2002–2023 in the continental United States. Tips colored to indicate observed breeding populations (black) vs. non-breeding populations (gray). Evidence for breeding based on: (1) confirmed or probable breeding code from eBird [52]; and (2) the criteria of Uehling and colleagues [30] in which there were at least twenty-five observations. Silhouettes are meant to signify major phylogenetic groups within Psittaciformes.

From the compiled lists of parrots sighted in the United States, we determined which species were established based on: (1) confirmed or probable breeding code from eBird [52]; and (2) the criteria of Uehling and colleagues [30] in which there were at least twenty-five observations. Following previously established protocols [56,57], we determined that there is a phylogenetic explanation for which species become established in the continental United States (Pagel’s lambda = 0.44, *p*-value = 0.007; Figure 2). Specifically, parrots in the family Cacatuidae and genus *Pyrrhura* appear to be poor at establishing themselves once released within the continental United States. Of course, such an analysis is cursory and there are many extenuating circumstances (e.g., time since first observation, number of original founding population, etc.) unaccounted for in our model that need to be considered prior to any definitive assertations.

Across the United States, naturalized parrot populations are primarily located in warm climates and/or urban areas (Figure 1). As of 2016, there were a reported fifty-six species of parrots observed in forty-three states with over 100,000 unique observations collected at ~19,000 unique localities. Of these, twenty-five species across twenty-three states are confirmed to have breeding populations [30]. Based on our updated analyses, as of 2023, seventy-three parrot species are observed in forty-seven of the contiguous United States (Figure 1). The observed rise in parrot species and corresponding records can be attributed to two primary factors. Firstly, the increase in parrot species may stem from a surge in the number of escapees. As more people keep parrots as pets, the likelihood of some individuals escaping or being released into the wild inadvertently increases. These escapees then contribute to the growing population of parrots in certain regions, leading to their inclusion in the records. Secondly, the increase in the use of eBird, a popular online platform for birdwatchers to submit their observations, has played a significant role in documenting parrot sightings. More birdwatchers are actively participating in recording their observations, including parrot species, through eBird. This wider engagement with the platform has led to a greater number of parrot records being reported and documented [52]. It is interesting to note that from 2016 to 2023, there has not been an increase in established breeding parrot species. Likely this is simply a reflection of the short sampling period between the two studies, but other factors may be at play that deserve future research. The three species accounting for most observations were the monk parakeet (*Myiopsitta monachus*), nanday conure (*Aratinga nenday*), and red-crowned amazon (*Amazona viridigenalis*).

### 1.3. Naturalized Parrots: A Legacy of the Pet Trade

In the United States and beyond, the presence of naturalized parrots is a direct consequence of the large-scale alteration of the landscape associated with the Anthropocene and the pet trade (both legal and illegal) [58]. Parrots are highly sought after for their strikingly colorful plumage, intelligence, and sociability, and have been pursued as companion animals for hundreds of years. Indeed, mummified and skeletonized remains for at least six species of parrots found in five archaeological sites in the Atacama Desert of northern Chile provides evidence that macaws, amazons, and conures were captured, kept, and transported by pre-Columbian Americans [59]. The continued demand for these rare animals as pets has allowed for this trade to be transformed into the lucrative business it is today [58]; indeed, parrots remain the most traded birds internationally [60,61,62]. The Convention on International Trade in Endangered Species (CITES) of Wild Fauna and Flora reported that 90% of all live birds traded were Psittaciformes, with more than 16 million parrots (representing 321 species) traded internationally between 1975 and 2016 [62], a figure that likely represents a gross underestimation as illegally traded parrots were not included in the count. The combination of natural habitat destruction in addition to unsustainable parrot poaching has led parrots to have the largest population of endangered species among birds worldwide [58,62]. The consequences of this demand are thought to have lasting ecological, economic, and ethical impacts.

Perhaps the most severe consequence is the global threat to ecological diversity. The Spix Macaw (*Cyanopsitta spixii*), a brilliantly blue parrot was declared extinct in the wild in 2020 and while a combination of threats (e.g., habitat loss, predators, climate change, disease) may have contributed to their disappearance, the illegal parrot trade undoubtedly had a substantial impact. The value of the rare Spix Macaw reached soaring prices of $20,000 per individual, further exacerbating the financial motivation to poach these animals [63].

The most recent population of parrots under enormous threat via trafficking are African grey parrots. Since 1992, Ghana has lost nearly 90–99% of its grey parrot population, a trend mirrored elsewhere in the continent where population estimates continue to plummet [64,65]. The collapse of these wild populations has united conservationists and animal activists with a common goal of reducing the impact of the parrot pet trade but, while such efforts have yielded some legislative impact, it remains unclear whether the implementation of more stringent conservation laws will reduce poaching [66].

Tellingly, among the ten most traded genera of parrot as reported by CITES between 1975 and 2016, five have well-established populations in the United States. These include lovebirds (*Agapornis*) in Arizona; monk parakeets (*Myiopsitta*) in Florida, New York, and Chicago; Amazon parrots (*Amazona*) in Florida and California; masked parakeets (*Psittacara*) in California; and South American conures (*Aratinga*) through central and southern Florida, some areas of California, and occasionally Arizona and Texas (Figure 1; [30,44]. While flourishing naturalized populations have served to increase population numbers, this upswing is dwarfed by the broader global decline in parrot populations and the metrics of overall parrot diversity.

### 1.4. What Makes Naturalized Parrots So Successful?

As outlined above, parrots represent a remarkable case study of urban success. Urban environments are key points for entry and establishment of exotic species, and recent work has demonstrated that non-native species richness is likely due to the rise in urban areas globally [67,68]. Urban environments are hotspots of human movement and transport of goods, which allows non-native species to become inadvertently established across long distances [36,37,69]. To this end, more than seventy-three species of exotic parrots have been reported across the continental United States, with 34% of these species establishing breeding populations (Table 1). One key question must be posed: to what should we attribute the remarkable success of parrots in these urban landscapes?

First, we must acknowledge the inherent variability and adaptability that defines parrots as a whole. Clearly, for any lineage to successfully colonize six continents, while occupying habitats that include both open desert and tropical rainforest, necessitates a high degree of behavioral flexibility [1,30,51]. Indeed, the dietary malleability of many parrot species allows them to find and exploit food resources across many different landscapes [1,43,70,71]. They demonstrate a wide range of food preferences, utilizing both native and non-native food sources [71,72,73]. By being able to adjust their diet based on available resources [71], parrots can successfully establish populations in new habitats and thrive in urban environments. Moreover, parrots show an extraordinary ability to shift their behaviors in response to novel environmental traits. This skill is encapsulated by a recent translocational study of yellow-naped Amazons (*Amazona auropalliata*), in which the newly released parrots rapidly altered their ranging patterns, habitat usage, and roosting behaviors to match resident birds at their release site, as opposed to maintaining the behavioral characteristics of their capture site [74]. Further support of behavioral flexibility is demonstrated by the positional behavior of naturalized monk parakeets in New York City. In this dense, urban environment, monk parakeets freely exploit many artificial substrates for perching and locomotion, including telephone wires, concrete (e.g., building facades), marble (e.g., gravestones), and metal (e.g., vehicles). Importantly, when using these artificial structures, monk parakeets adopted a more diverse locomotor repertoire [57] with less reliance on a single locomotor modality [32]. Accordingly, locomotor diversity was higher when moving on artificial structures compared to natural substrates, highlighting their behavioral flexibility in response to human-mediated environmental change [32]. Through this lens, then, it is perhaps unsurprising that parrots are so adept at urban and suburban colonization.

A second clear point in favor of naturalized parrots is their widespread public appeal. For much of the general public, the notion of an “invasive” species infiltrating their community stirs up feelings of vague, shadowy creatures outside the common cultural zeitgeist [43]. From black rats to cane toads, such animals are classically recalled as the augurs of widespread ecological disaster. Contrast this to the almost comical, but certainly endearing visual scene of urban parrots: whose enigmatic, colorful charisma brightens the landscape and lends itself readily to photographic opportunities [43,75]. In this context, many communities have embraced naturalized parrot populations as another “fresh face” within the neighborhood, imbuing local parks, gardens, and beaches with a sense of tropical style. Often, these populations become forms of “community pets”—shared and enjoyed by both locals and tourists alike, while free from the traditional responsibilities of animal husbandry [75].

Ironically, however, perhaps the most critical aspect underlying the success of naturalized parrots is the same trait that first led to their popularity as a human companion: their cognitive ability. The problem-solving abilities of parrots are well-documented; indeed, as early as the 1920s, parrots were earmarked as outperforming other avians in numerical assessment tasks [76,77]. More recently, experiments with trained African grey parrots have demonstrated their ability to categorize unknown objects, grasp abstract concepts such as smaller vs. larger, and utilize a vocabulary in excess of a hundred words [78,79,80]. Similarly, recent work on cockatiel cognition has demonstrated their previously underappreciated aptitude for referential communication and impulse control when participating in group tasks [77], while Goffin’s cockatoos have demonstrated the ability to innovatively use tools to solve sophisticated spatial problems [81,82]. Indeed, not only are parrots strong positive outliers among vertebrates in their relative brain sizes [83,84,85,86], but they possess a neuron density almost two-times that of mammals with similarly sized brains [87]. Thus, in many regards it has been suggested that the brains of parrots functionally resemble those of higher primates [55,77,87,88,89].

**Figure 3:**
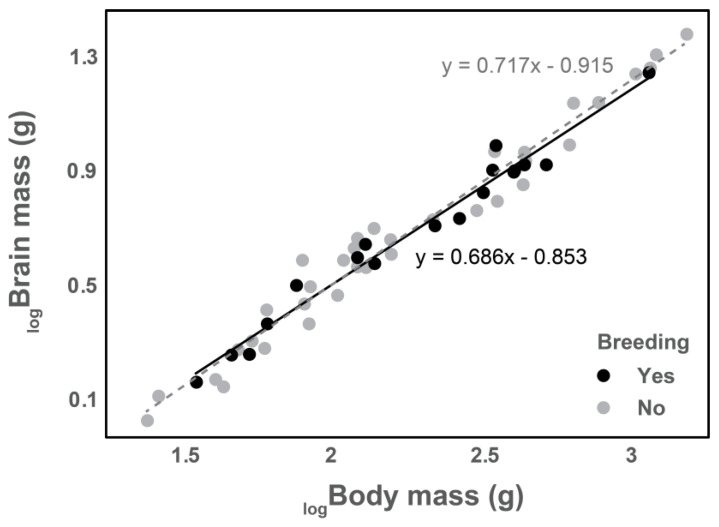
Bivariate regression of the relationship between body mass and brain mass in naturalized US parrots. Data divided between US breeding (black; *y* = 0.686x − 0.853) vs. non-breeding species (grey; *y* = 0.717x − 0.915). All data log-transformed. We observed no evidence (*p* = 0.341) to suggest that relatively big-brained parrots are more likely to establish breeding populations once released in the United States. Data on brain and body mass collected from Iwaniuk [55].

The association between intelligence/behavioral flexibility and the ability for a species to become established in a novel environment is not a new assertion. Using a phylogenetic generalized least squares (PGLS) regression [90,91], we tested whether relative brain size (data on brain size from [55]), an imperfect but commonly used metric for intelligence, is indicative of what parrot species observed in the continental United States become established breeders (Figure 2). Additional information on body mass and original observation date is included in the PGLS model. This analysis was performed in *R* [54] using the packages “phytools” [92], “tidyr” [93], and “dplyr” [94]. We observed no evidence (*p* = 0.341) to suggest that relatively big-brained parrots are more likely to establish breeding populations in the United States (Figure 3). Similarly, there was no effect (*p* = 0.381) of body mass (Figure 3). The only significant factor (*p* = 0.002) that determines whether a parrot species is likely to become established is the time since first introduced. Thus, from a management perspective, all efforts should be made to locate and eradicate naturalized parrot populations as soon as they are introduced if the goal is to prevent establishment. This is a cursory model and additional information concerning other factors (e.g., original population size, local temperature, reproductive rate, etc.) should be considered before any definitive assertations can be made.

## 2. Conclusions

Due to their charismatic and colorful nature, parrots have had a long history as human companions. Accordingly, parrots have become a casualty of the pet trade and while native populations have decreased globally, there have been multiple naturalized introductions. Within the continental United States there are currently seventy-three species of introduced parrot species observed across forty-eight states (Figure 1), a number that has increased since the 2016 survey conducted by Uehling and colleagues [1]. We determined that the likelihood of an introduced species becoming established is phylogenetically driven, with members of the family Cacatuidae and *Pyrrhura* being particularly bad at establishing breeding populations once being released (Figure 2). Relative brain size and body mass within parrots is a poor predictor of whether a species becomes established or not. We suggest that the success of parrots as naturalized species is due in part to their charismatic nature and overall behavioral flexibility. Future work should be conducted to assess how parrot species adapt behaviorally to their surroundings once being introduced to novel areas.

## Figures and Tables

**Table 1:** Naturalized parrot species observed in the contiguous United States, whether there is evidence for breeding, body mass, brain mass, and relative brain size. Evidence for breeding based on: (1) confirmed or probable breeding code from eBird [52]; and (2) the criteria of Uehling and colleagues [30] in which there were at least twenty-five observations. Data on brain and body mass collected from Iwaniuk [55].

Species	Evidence for Breeding	Body Mass (g)	Brain Mass (g)	Relative Brain (%)	Species	Evidence for Breeding	Body Mass (g)	Brain Mass (g)	Relative Brain (%)
*Agapornis fischeri*	No	48.30	1.95	4.04	*Eupsittula aurea*	No	84.00	3.23	3.85
*Agapornis lilianae*	No	40.50	1.53	3.78	*Eupsittula canicularis*	No	75.20	3.26	4.34
*Agapornis personatus*	Yes	52.50	1.87	3.56	*Eupsittula nana*	No	79.00	4.01	5.08
*Agapornis pullarius*	No	43.00	1.44	3.35	*Forpus coelestis*	No	26.20	1.34	5.11
*Agapornis roseicollis*	Yes	45.80	1.86	4.06	*Forpus passerinus*	No	24.00	1.10	4.58
*Amazona aestiva*	Yes	400.00	8.24	2.06	*Forpus xanthops*	No	-	-	-
*Amazona albifrons*	Yes	218.00	5.28	2.42	*Melopsittacus undulatus*	Yes	35.00	1.50	4.29
*Amazona amazonica*	Yes	338.00	8.29	2.45	*Myiopsitta monachus*	Yes	120.00	4.08	3.40
*Amazona auropalliata*	No	433.00	9.57	2.21	*Nymphicus hollandicus*	No	83.00	2.39	2.88
*Amazona autumnalis*	Yes	399.50	8.13	2.04	*Pionites melanocephalus*	No	136.40	5.18	3.80
*Amazona farinosa*	No	610.00	10.14	1.66	*Pionus maximiliani*	No	263.00	5.60	2.13
*Amazona finschi*	Yes	-	-	-	*Pionus senilis*	No	-	-	-
*Amazona ochrocephala*	Yes	510.00	8.64	1.69	*Platycercus elegans*	No	128.60	3.78	2.94
*Amazona oratrix*	Yes	433.00	8.62	1.99	*Platycercus eximius*	No	103.50	3.01	2.91
*Amazona ventralis*	No	300.00	5.97	1.99	*Poicephalus flavifrons*	No	-	-	-
*Amazona viridigenalis*	Yes	316.00	6.89	2.18	*Poicephalus meyeri*	No	117.50	4.41	3.75
*Amazona xantholora*	No	-	-	-	*Poicephalus senegalus*	No	155.00	4.71	3.04
*Anodorhynchus hyacinthinus*	No	1500.00	24.73	1.65	*Psephotus haematonotus*	No	59.20	1.97	3.33
*Ara ararauna*	Yes	1125.00	18.08	1.61	*Psittacara erythrogenys*	Yes	-	-	-
*Ara chloropterus*	No	1185.00	20.88	1.76	*Psittacara finschi*	Yes	-	-	-
*Ara macao*	No	1015.00	17.93	1.77	*Psittacara holochlorus*	Yes	-	-	-
*Ara militaris*	No	1134.00	18.83	1.66	*Psittacara leucophthalmus*	Yes	-	-	-
*Ara severus*	Yes	347.00	10.05	2.90	*Psittacara mitratus*	Yes	-	-	-
*Aratinga jandaya*	No	120.00	3.80	3.17	*Psittacara strenuus*	No	-	-	-
*Aratinga nenday*	Yes	128.00	4.55	3.55	*Psittacara wagleri*	Yes	-	-	-
*Aratinga weddellii*	No	108.00	4.01	3.71	*Psittacula alexandri*	No	156.00	4.20	2.69
*Bolborhynchus lineola*	No	53.60	2.08	3.88	*Psittacula cyanocephala*	No	60.00	2.68	4.47
*Brotogeris chiriri*	Yes	-	-	-	*Psittacula eques*	No	-	-	-
*Brotogeris versicolurus*	Yes	60.40	2.39	3.96	*Psittacula eupatria*	No	214.00	5.54	2.59
*Cacatua alba*	No	631.00	14.16	2.24	*Psittacula krameri*	Yes	137.00	3.90	2.85
*Cacatua galerita*	No	765.00	14.24	1.86	*Psittacus erithacus*	No	-	-	-
*Cacatua sanguinea*	No	437.50	8.91	2.04	*Pyrrhura frontalis*	No	80.10	2.81	3.51
*Cacatua sulphurea*	No	344.00	9.62	2.80	*Pyrrhura molinae*	No	-	-	-
*Cyanoliseus patagonus*	No	-	-	-	*Pyrrhura rupicola*	No	-	-	-
*Eclectus roratus*	No	428.00	7.36	1.72	*Thectocercus acuticaudatus*	Yes	-	-	-
*Eolophus roseicapilla*	No	351.00	6.43	1.83	*Trichoglossus moluccanus*	No	-	-	-
*Eos bornea*	No	120.00	4.78	3.98					

## Data Availability

All data used for statistical analysis within this study are presented in [52,55].

## References

[1] del Hoyo J., Elliott A., Sargatal J., Christie D.A. (2010). Handbook of the Birds of the World.

[2] Gupta S.N., Cruz M.S., Maqsood F., Granatosky M.C., Vonk J., Shackelford T. (2020). Psittaciformes Locomotion. Encyclopedia of Animal Cognition and Behavior.

[3] Price-Waldman R., Stoddard M.C. (2021). Avian Coloration Genetics: Recent Advances and Emerging Questions. J. Hered..

[4] Marler P. (2004). Bird Calls: Their Potential for Behavioral Neurobiology. Ann. N. Y. Acad. Sci..

[5] Munshi-South J., Wilkinson G.S. (2006). Diet Influences Life Span in Parrots (Psittaciformes). Auk.

[6] Zeigler H.P. (1975). Some Observations on the Development of Feeding in Captive Kea (Nestor Notabilis). Notornis.

[7] Diamond J., Bond A.B. (1999). Kea, Bird of Paradox: The Evolution and Behavior of a New Zealand Parrot.

[8] Wright T.F., Schirtzinger E.E., Matsumoto T., Eberhard J.R., Graves G.R., Sanchez J.J., Capelli S., Müller H., Scharpegge J., Chambers G.K. (2008). A Multilocus Molecular Phylogeny of the Parrots (Psittaciformes): Support for a Gondwanan Origin during the Cretaceous. Mol. Biol. Evol..

[9] Provost K.L., Joseph L., Smith B.T. (2018). Resolving a Phylogenetic Hypothesis for Parrots: Implications from Systematics to Conservation. Emu.—Austral. Ornithol..

[10] Suh A., Paus M., Kiefmann M., Churakov G., Franke F.A., Brosius J., Kriegs J.O., Schmitz J. (2011). Mesozoic Retroposons Reveal Parrots as the Closest Living Relatives of Passerine Birds. Nat. Commun..

[11] Mayr G. (2010). Parrot Interrelationships—Morphology and the New Molecular Phylogenies. Emu.

[12] Mayr G. (2014). The Origins of Crown Group Birds: Molecules and Fossils. Palaeontology.

[13] Waterhouse D.M. (2006). Parrots in a Nutshell: The Fossil Record of Psittaciformes (Aves). Hist. Biol..

[14] Tavares E.S., Baker A.J., Pereira S.L., Miyaki C.Y. (2006). Phylogenetic Relationships and Historical Biogeography of Neotropical Parrots (Psittaciformes: Psittacidae: Arini) Inferred from Mitochondrial and Nuclear DNA Sequences. Syst. Biol..

[15] Waterhouse D.M., Lindow B.E.K., Zelenkov N.V., Dyke G.J. (2008). Two New Parrots (Psittaciformes) from the Lower Eocene Fur Formation of Denmark. Palaeontology.

[16] Mayr G., Bertelli S. (2011). A Record of Rhynchaeites (Aves, Threskiornithidae) from the Early Eocene Fur Formation of Denmark, and the Affinities of the Alleged Parrot Mopsitta. Palaeobiodiv. Palaeoenviron..

[17] Ksepka D.T., Clarke J.A. (2012). A New Stem Parrot from the Green River Formation and the Complex Evolution of the Grasping Foot in Pan-Psittaciformes. J. Vertebr. Paleontol..

[18] Joseph L., Toon A., Schirtzinger E.E., Wright T.F., Schodde R. (2012). A Revised Nomenclature and Classification for Family-Group Taxa of Parrots (Psittaciformes). Zootaxa.

[19] Holdaway R.N., Worthy T.H. (1993). First North Island Fossil Record of Kea, and Morphological and Morphometric Comparison of Kea and Kaka. Notornis.

[20] White N.E., Phillips M.J., Gilbert M.T.P., Alfaro-Núñez A., Willerslev E., Mawson P.R., Spencer P.B.S., Bunce M. (2011). The Evolutionary History of Cockatoos (Aves: Psittaciformes: Cacatuidae). Mol. Phylogenet. Evol..

[21] Bergtold W.H. (1906). Concerning the Thick-Billed Parrot. The Auk.

[22] Snyder N.F.R., Koenig S.E., Koschmann J., Snyder H.A., Johnson T.B. (1994). Thick-Billed Parrot Releases in Arizona. The Condor.

[23] Monterrubio-Rico T., Enkerlin-Hoeflich E. (2004). Present Use and Characteristics of Thick-Billed Parrot Nest Sites in Northwestern Mexico. J. Field Ornithol..

[24] Saikku M. (1990). The Extinction of the Carolina Parakeet. Environ. Hist. Rev..

[25] Elphick C.S., Roberts D.L., Michael Reed J. (2010). Estimated Dates of Recent Extinctions for North American and Hawaiian Birds. Biol. Conserv..

[26] Kirchman J.J., Schirtzinger E.E., Wright T.F. (2012). Phylogenetic Relationships of the Extinct Carolina Parakeet (Conuropsis Carolinensis) Inferred from DNA Sequence Data. Auk.

[27] Burgio K.R., Carlson C.J., Tingley M.W. (2017). Lazarus Ecology: Recovering the Distribution and Migratory Patterns of the Extinct Carolina Parakeet. Ecol. Evol..

[28] Gelabert P., Sandoval-Velasco M., Serres A., de Manuel M., Renom P., Margaryan A., Stiller J., de-Dios T., Fang Q., Feng S. (2020). Evolutionary History, Genomic Adaptation to Toxic Diet, and Extinction of the Carolina Parakeet. Curr. Biol..

[29] Frei B., Smith K.G., Withgott J.H., Rodewald P.G., Pyle P., Patten M.A., Rodewald P.G. (2015). Red-Headed Woodpecker (Melanerpes erythrocephalus). The Birds of North America.

[30] Uehling J.J., Tallant J., Pruett-Jones S. (2019). Status of Naturalized Parrots in the United States. J. Ornithol..

[31] Butler C.J. (2005). Feral Parrots in the Continental United States and United Kingdom: Past, Present, and Future. J. Avian Med. Surg..

[32] Granatosky M.C., Young M.W., Herr V., Chai C., Raidah A., Kairo J.N., Anaekwe A., Havens A., Zou B., Ding B. (2022). Positional Behavior of Introduced Monk Parakeets (Myiopsitta Monachus) in an Urban Landscape. Animals.

[33] Pruett-Jones S. (2021). Naturalized Parrots of the World: Distribution, Ecology, and Impacts of the World’s Most Colorful Colonizers.

[34] Cassey P., Blackburn T.M., Russell G.J., Jones K.E., Lockwood J.L. (2004). Influences on the Transport and Establishment of Exotic Bird Species: An Analysis of the Parrots (Psittaciformes) of the World. Glob. Change Biol..

[35] Blackburn T.M., Pyšek P., Bacher S., Carlton J.T., Duncan R.P., Jarošík V., Wilson J.R.U., Richardson D.M. (2011). A Proposed Unified Framework for Biological Invasions. Trends Ecol. Evol..

[36] Myers J.H., Simberloff D., Kuris A.M., Carey J.R. (2000). Eradication Revisited: Dealing with Exotic Species. Trends Ecol. Evol..

[37] Krysko K., Burgess J., Rochford M., Gillette C.R., Cueva D., Enge K.M., Somma L.A., Stabile J.L., Smith D.C., Wasilewski J.A. (2011). Verified Non-Indigenous Amphibians and Reptiles in Florida from 1863 through 2010: Outlining the Invasion Process and Identifying Invasion Pathways and Stages. Zootaxa.

[38] Temple S.A. (1992). Exotic Birds: A Growing Problem with No Easy Solution. Auk.

[39] Kessel B. (1953). Distribution and Migration of the European Starling in North America. Condor.

[40] Linz G.M., Homan H.J., Gaulker S.M., Penry L.B., Bleier W.J. (2007). European starlings: A review of an invasive species with far-reaching impacts. Manag. Vertebr. Invasive Species.

[41] Markula A., Hannan-Jones M., Csurhes S. (2009). Pest Animal Risk Assessment: Indian Myna, Acridotheres Tristis. Pest Anim. Risk Assess. Indian Myna Acridotheres Tristis.

[42] Rogers A.M., Griffin A.S., van Rensburg B.J., Kark S. (2020). Noisy Neighbours and Myna Problems: Interaction Webs and Aggression around Tree Hollows in Urban Habitats. J. Appl. Ecol..

[43] Baldwin S.C. (2015). The Brooklyn Parrots FAQ: All about the Wild Monk Parakeets of Brookyn, NY.

[44] Calzada Preston C.E., Pruett-Jones S. (2021). The Number and Distribution of Introduced and Naturalized Parrots. Diversity.

[45] Vall-llosera M., Cassey P. (2017). Leaky Doors: Private Captivity as a Prominent Source of Bird Introductions in Australia. PLoS ONE.

[46] S.F. Moves to Make Wild Parrots the Official Animal of the City. https://www.sfchronicle.com/sf/bayarea/heatherknight/article/sf-official-animal-total-sf-wild-parrots-17878938.php.

[47] S.F. Has a New Official Animal. Long May the Wild Parrots Fly!. https://www.sfchronicle.com/bayarea/article/official-animal-wild-parrot-17869732.php.

[48] Castro J., Sáez C., Molina-Morales M. (2022). The Monk Parakeet (Myiopsitta Monachus) as a Potential Pest for Agriculture in the Mediterranean Basin. Biol. Invasions.

[49] Davis L.R. (1974). The Monk Parakeet: A Potential Threat to Agriculture. Proc. Vertebr. Pest Conf..

[50] Avery M.L., Greiner E.C., Lindsay J.R., Newman J.R., Pruett-Jones S. (2002). Monk Parakeet Management at Electric Utility Facilities in South Florida. Proc. Vertebr. Pest Conf..

[51] Kiacz S., Pruett-Jones S. (2021). Brightsmith Naturalized Parrots: Conservation and Research Opportunites. Naturalized Parrots of the World: Distribution, Ecology, and Impacts of the World’s Most Colorful Colonizers.

[52] eBird EBird: An Online Database of Bird Distribution and Abundance [Web Application]. https://science.ebird.org/en/use-ebird-data/citation.

[53] Kumar S., Suleski M., Craig J.M., Kasprowicz A.E., Sanderford M., Li M., Stecher G., Hedges S.B. (2022). TimeTree 5: An Expanded Resource for Species Divergence Times. Mol. Biol. Evol..

[54] R Core Team (2021). R: A Language and Environment for Statistical Computing.

[55] Iwaniuk A.N., Dean K.M., Nelson J.E. (2004). Interspecific Allometry of the Brain and Brain Regions in Parrots (Psittaciformes): Comparisons with Other Birds and Primates. Brain. Behav. Evol..

[56] Münkemüller T., Lavergne S., Bzeznik B., Dray S., Jombart T., Schiffers K., Thuiller W. (2012). How to Measure and Test Phylogenetic Signal. Methods Ecol. Evol..

[57] Granatosky M.C. (2018). A Review of Locomotor Diversity in Mammals with Analyses Exploring the Influence of Substrate-Use, Body Mass, and Intermembral Index in Primates. J. Zool..

[58] Pires S.F. (2012). The Illegal Parrot Trade: A Literature Review. Glob. Crime.

[59] Capriles J.M., Santoro C.M., George R.J., Flores Bedregal E., Kennett D.J., Kistler L., Rothhammer F. (2021). Pre-Columbian Transregional Captive Rearing of Amazonian Parrots in the Atacama Desert. Proc. Natl. Acad. Sci. USA..

[60] Bush E.R., Baker S.E., Macdonald D.W. (2014). Global Trade in Exotic Pets 2006–2012. Conserv. Biol..

[61] Furnell S. (2019). Strengthening CITES Processes for Reviewing Trade in Captive-Bred Specimens and Preventing Mis-Decla.

[62] Chan D.T.C., Poon E.S.K., Wong A.T.C., Sin S.Y.W. (2021). Global Trade in Parrots—Influential Factors of Trade and Implications for Conservation. Glob. Ecol. Conserv..

[63] Wright T.F., Toft C.A., Enkerlin-Hoeflich E., Gonzalez-Elizondo J., Albornoz M., Rodríguez-Ferraro A., Rojas-Suárez F., Sanz V., Trujillo A., Beissinger S.R. (2001). Nest Poaching in Neotropical Parrots. Conserv. Biol..

[64] Annorbah N.N.D., Collar N.J., Marsden S.J. (2016). Trade and Habitat Change Virtually Eliminate the Grey Parrot Psittacus Erithacus from Ghana. Ibis.

[65] Martin R.O., Senni C., D’Cruze N.C. (2018). Trade in Wild-Sourced African Grey Parrots: Insights via Social Media. Glob. Ecol. Conserv..

[66] Rowcliffe J.M., de Merode E., Cowlishaw G. (2004). Do Wildlife Laws Work? Species Protection and the Application of a Prey Choice Model to Poaching Decisions. Proc. R. Soc. Lond. B Biol. Sci..

[67] Munyenyembe F., Harris J., Hone J., Nix H. (1989). Determinants of Bird Populations in an Urban Area. Aust. J. Ecol..

[68] Vidra R.L., Shear T.H., Wentworth T.R. (2006). Testing the Paradigms of Exotic Species Invasion in Urban Riparian Forests. Nat. Areas J..

[69] Palmer M.W. (2005). Temporal Trends of Exotic Species Richness in North American Floras: An Overview. Écoscience.

[70] Álvarez-Castillo C., MacGregor-Fors I., Arriaga-Weiss S.L., Mota-Vargas C., Santiago-Alarcon D. (2022). Abundance of White-Fronted Parrots and Diet of an Urban Parrot Assemblage (Aves: Psittaciformes) in a Green Neotropical City. Avian Res..

[71] South J.M., Pruett-Jones S. (2000). Patterns of Flock Size, Diet, and Vigilance of Naturalized Monk Parakeets in Hyde Park, Chicago. Condor.

[72] Mabb K.T., Collins C.T., Kares L.M. (1997). Food items of naturalized parrots in southern calwornia. West. Birds.

[73] Martens J., Hoppe D., Woog F. (2013). Diet and Feeding Behaviour of Naturalised Amazon Parrots in a European City. Ardea.

[74] Salinas-Melgoza A., Salinas-Melgoza V., Wright T.F. (2013). Behavioral Plasticity of a Threatened Parrot in Human-Modified Landscapes. Biol. Conserv..

[75] Crowley S.L., Pruett-Jones S. (2021). Parrots and People: Human Dimensions of Naturalized Parrots. Naturalized Parrots of the World: Distribution, Ecology, and Impacts of the World’s Most Colorful Colonizers.

[76] Fischel W. (1926). Zeitschrift Für Vergleichende Physiologie. Z Vergl Physiol.

[77] Auersperg A.M.I., Bayern A.M.P. (2019). von Who’s a Clever Bird—Now? A Brief History of Parrot Cognition. Behaviour.

[78] Pepperberg I.M. (2002). Cognitive and Communicative Abilities of Grey Parrots. Curr. Dir. Psychol. Sci..

[79] Vick S.-J., Bovet D., Anderson J.R. (2010). How Do African Grey Parrots (Psittacus Erithacus) Perform on a Delay of Gratification Task?. Anim. Cogn..

[80] Péron F., Rat-Fischer L., Lalot M., Nagle L., Bovet D. (2011). Cooperative Problem Solving in African Grey Parrots (Psittacus Erithacus). Anim. Cogn..

[81] Auersperg A.M.I., Köck C., Pledermann A., O’Hara M., Huber L. (2017). Safekeeping of Tools in Goffin’s Cockatoos, Cacatua Goffiniana. Anim. Behav..

[82] Laumer I.B., Bugnyar T., Auersperg A.M.I. (2016). Flexible Decision-Making Relative to Reward Quality and Tool Functionality in Goffin Cockatoos (Cacatua Goffiniana). Sci. Rep..

[83] Smeele S.Q., Conde D.A., Baudisch A., Bruslund S., Iwaniuk A., Staerk J., Wright T.F., Young A.M., McElreath M.B., Aplin L. (2022). Coevolution of Relative Brain Size and Life Expectancy in Parrots. Proc. R. Soc. B.

[84] Schuck-Paim C., Alonso W.J., Ottoni E.B. (2008). Cognition in an Ever-Changing World: Climatic Variability Is Associated with Brain Size in Neotropical Parrots. Brain. Behav. Evol..

[85] Ksepka D.T., Balanoff A.M., Smith N.A., Bever G.S., Bhullar B.-A.S., Bourdon E., Braun E.L., Burleigh J.G., Clarke J.A., Colbert M.W. (2020). Tempo and Pattern of Avian Brain Size Evolution. Curr. Biol..

[86] Andrews C.B., Gregory T.R. (2009). Genome Size Is Inversely Correlated with Relative Brain Size in Parrots and Cockatoos. Genome.

[87] Olkowicz S., Kocourek M., Lučan R.K., Porteš M., Fitch W.T., Herculano-Houzel S., Němec P. (2016). Birds Have Primate-like Numbers of Neurons in the Forebrain. Proc. Natl. Acad. Sci. USA.

[88] Gutiérrez-Ibáñez C., Iwaniuk A.N., Wylie D.R. (2018). Parrots Have Evolved a Primate-like Telencephalic-Midbrain-Cerebellar Circuit. Sci. Rep..

[89] Krasheninnikova A., Berardi R., Lind M.A., O’Neill L., von Bayern A.M. (2019). Primate Cognition Test Battery in Parrots. Behaviour.

[90] Revell L.J. (2010). Phylogenetic Signal and Linear Regression on Species Data. Methods Ecol. Evol..

[91] Mundry R., Garamszegi L.Z. (2014). Statistical Issues and Assumptions of Phylogenetic Generalized Least Squares. Modern Phylogenetic Comparative Methods and Their Application in Evolutionary Biology: Concepts and Practice.

[92] Revell L.J. (2012). Phytools: An R Package for Phylogenetic Comparative Biology (and Other Things). Methods Ecol. Evol..

[93] Mailund T., Mailund T. (2022). Reformatting Tables: Tidyr. R 4 Data Science Quick Reference: A Pocket Guide to APIs, Libraries, and Packages.

[94] Wickman H., François R., Henry L., Müller K. (2018). Dplyr: A Grammar of Data Manipulation, Version 0.7. 7 2018.

